# Inhibition of GSK-3β Restores Differentiation Potential of Late-Passage Mesenchymal Stem Cells

**DOI:** 10.3390/ph18040483

**Published:** 2025-03-28

**Authors:** Kavitha Govarthanan, Raja Sundari Meenakshi Sundaram, Arthi Sunil Richard, Siva Chander Chabathula, Secunda Rupert, Jeswanth Sathyanesan, Rama Shanker Verma, Naveen Jeyaraman, Madhan Jeyaraman, Ramya Lakshmi Rajendran, Prakash Gangadaran, Byeong-Cheol Ahn

**Affiliations:** 1 Department of Biotechnology, Indian Institute of Technology Madras, Chennai 600036, Tamil Nadu, India; govarthanan_kavitha@yahoo.co.in (K.G.); arthihard@gmail.com (A.S.R.); siva.chabathula@gmail.com (S.C.C.); 2 Centre for Cardiovascular Biology and Disease, Institute for Stem Cell Sciences and Regenerative Medicine, Bengaluru 560065, Karnataka, India; 3 Department of Regenerative Medicine and Research, Government Stanley Hospital, Chennai 600001, Tamil Nadu, India; bt.raji@gmail.com (R.S.M.S.); drsecunda@gmail.com (S.R.); dr_jeswanth@yahoo.co.in (J.S.); 4 Department of Orthopaedics, ACS Medical College and Hospital, Dr MGR Educational and Research Institute, Chennai 600077, Tamil Nadu, India; naveenjeyaraman@yahoo.com (N.J.); madhanjeyaraman@gmail.com (M.J.); 5 BK21 FOUR KNU Convergence Educational Program of Biomedical Sciences for Creative Future Talents, Department of Biomedical Sciences, School of Medicine, Kyungpook National University, Daegu 41944, Republic of Korea; ramyag@knu.ac.kr; 6 Department of Nuclear Medicine, School of Medicine, Kyungpook National University, Daegu 41944, Republic of Korea; 7 Cardiovascular Research Institute, Kyungpook National University, Daegu 41944, Republic of Korea; 8 Department of Nuclear Medicine, Kyungpook National University Hospital, Daegu 41944, Republic of Korea

**Keywords:** MSCs, senescence, rejuvenation, CHIR 99021, tri-lineage, differentiation

## Abstract

**Background/Objectives:** Mesenchymal stem cells (MSCs) are regarded as a promising cell type with significant therapeutic benefits owing to their ease of isolation, maintenance, and characterisation. However, repeated passages during cultural maintenance frequently result in cellular senescence, limiting their utility in regenerative medicine. **Methods:** We investigated the differentiation capability between early- (P3) and late-passage MSCs (>P15) and tested the potential of Wnt agonist 99021 to reverse MSCs using standard cell culture protocols that define minimal criteria for MSCs, primarily tri-lineage differentiation assays, biochemical staining gene expression analysis, and senescence assays. **Results:** We initially noticed distinct signs of morphological aging between early- (P3) and late-passage MSCs (>P15) and further examined the differentiation capability between early- (P3) and late-passage MSCs (>P15). We found a diminished differentiation potential in late-passage MSCs. Our senescence assay also revealed >P15 cells were able to absorb the senescence dye, indicating that >P15 MSCs underwent senescence. We further demonstrated that CHIR 99021 reversed the differentiation inhibitory potential-mediated impasse of late-passage MSCs by employing tri-lineage specific differentiation assays, biochemical labelling, and gene expression analysis. Senescence assays after CHIR 99021 treatment also revealed no senescence dye uptake at all. **Conclusions:** Our findings demonstrated that CHIR 99021 Wnt agonist maybe aids in the reversal of MSC aging-related differentiation inhibition glitches and offers a proven demonstrated protocol for rejuvenating late-passage MSCs. Thus, CHIR99021 treatment inherently reverts the tri-lineage potency in late-passage MSCs, and this method could be further employed to ensure a plentiful MSC source for clinical purposes.

## 1. Introduction

Cell-based therapeutic applications have attained immense attention and are being considered for treating metabolic and diseased conditions in clinical settings. Stem cells derived from allogenic sources are currently exploited in cell-based regenerative approaches for better disease management [1,2,3]. Mesenchymal stem cells (MSCs) from extra-embryonic and adult sources are frequently employed for the aforesaid approaches pertaining to their potency and immune-modulatory nature. The potency of MSCs is restricted to differentiate into certain specific lineages, but they do so with a far greater fidelity and specificity than ESCs or iPSCs [4]. Recent studies seeking the identification of cues exerting the maximum MSC differentiation potential demonstrate the hopes of gaining unlimited enhanced potential populations of MSCs, however, late-passage MSCs failed to engraft and ameliorate diseased conditions [5,6,7,8].

Abundant numbers of homogenous cells are vital for their application in clinical settings, and this could be achieved by upscaling MSCs under in vitro culture conditions. This often requires the recurrent passaging of MSCs, maintaining the stable blueprint of genomic and epigenomic landscapes in order to achieve the appropriate clinical dosage level [9]. It is well known that MSCs from various sources have distinct characteristic features such as proliferative potential, differentiation tendency, and secretory profiles [3,10,11]. The cellular event of aging/senescence typically leads to slowed replication and the exhaustion of differentiation potential in MSCs under recurrent in vitro passaging conditions [12,13,14,15]. The aforementioned hallmarks of senescence are major impediments, restricting the sourcing of MSCs and limiting their clinical applications.

MSC replicative senescence can be identified by its manifestation of distinct characteristic alterations. It includes induced structural changes such as the appearance of stress fibres, loss of typical morphology, and functionally compromised differentiation potential after being passaged for a longer time [16,17,18]. It is also observed that the secretory profiling of MSCs is also reduced, therefore, late-passage MSCs are drastically impaired with a loss of regenerative potential and are unsuitable for clinical applications [19]. The current concern of addressing MSC aging could be achieved either by limiting their proliferation potential or by rescuing or reversing MSC aging using efficient small-molecule inhibitors. Therefore, attempting research on the rejuvenation of MSCs will ease their hindrances and broaden the sources of MSCs that can be used for regenerative therapeutic purposes.

Earlier studies confirmed that WNT signals promoted the growth and developmental ability of MSCs and embryonic progenitors under extended culture conditions [20,21]. Moreover, the WNT/-catenin signalling pathway is well known for sustaining the self-renewing property in numerous types of stem cells, including embryonic stem cells [22], epidermal stem cells from hair follicles [23], and intestinal stem cells [24]. Therefore, it is well understood that the WNT/-catenin signalling pathway facilitates the proliferation of the stem cells while stalling their differentiation induction. A recent study also demonstrated that by supplementing a WNT ligand, WNT3A augmented MSC proliferation and developmental potential by shielding the cells from the harmful consequences of senescence rather than by controlling proliferation and differentiation [25]. Amongst the various reported Wnt agonists, CHIR 99021 is considered to be unique due to its specificity in targeting only GSK3beta subunits compared to other inhibitors that exhibit CDK inhibitory off-target effects [26]. Additionally, our earlier study also demonstrated that the WNT agonist CHIR 99021 played a crucial role in pre-tuning Wharton’s jelly-derived MSCs into a more potent state of enhanced differentiation via exhibiting primitive markers such as OCT4, SOX2, Nanog, and c-Myc, respectively [4].

Although the mechanisms behind MSC senescence are still not fully understood, studies have shown the elements of age-related MSC phenotypic alterations and their potential underlying mechanisms. It is vital to understand the morphological, biological, and stem cell marker alterations incurred during MSC aging [12,13,14]. Elucidating the cellular and molecular mechanisms that underlie MSC senescence may shed light on identifying innovative techniques or methodologies to rejuvenate senescent MSCs, thereby combating MSC aging. Given the background prerequisite for MSC rejuvenation strategies, in this current study, we investigated the role of CHIR 99021 in rejuvenating the aging of MSCs under in vitro culture conditions. Here, we were interested in analysing the impact of CHIR 99021 in rescuing tri-lineage differentiation potential, mainly in late-passage umbilical cord Wharton’s jelly-derived MSCs (UC-WJ-MSCs), thus capitalising on this technique for clinical settings.

## 2. Results

### Isolation of Mesenchymal Stem Cells from Wharton’s Jelly (WJ-MSCs)

**Figure 1:**
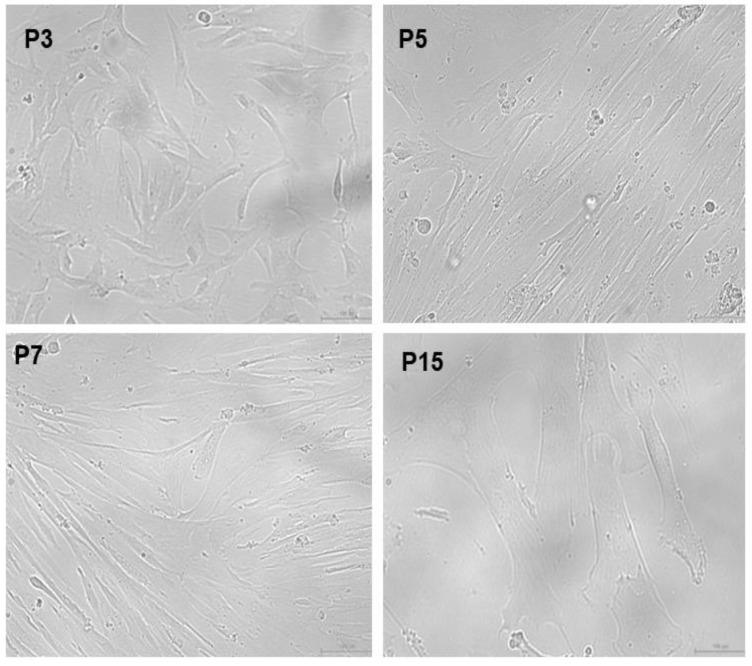
Bright-field microscopic image of WJ-MSCs showing aging related features. P3 WJ-MSCs showing typical phenotype of MSCs. P5 WJ-MSCs showing negligible features of stress fibres. P7 WJ-MSCs showing heterogenous morphological features suggested to undergo age-related stress. P15 WJ-MSCs showing enlarged morphology, with appearance of stress fibres (scale bar—10 µM).

Human MSCs (hMSCs) were successfully isolated from umbilical cord tissue derived from the Wharton’s jelly region. The isolated MSCs showed cultural properties like adherence to tissue culture plates, exhibiting typical fibroblast-like morphology (Figure 1, P3, P5). The isolated populations abundantly expressed more than 95% of CD105, CD90, and CD44 markers on their surface, whereas negligible expressions of CD34 and CD45 markers (<2%) were observed [4].

The MSCs were passaged by using 0.25% trypsin enzyme upon reaching 80 to 90% confluency. We observed MSCs from early passages until P5 (Figure 1, top panel), showing a typical pre-requisite morphological characteristic, however, upon subsequent passaging, these MSCs began to lose their typical fibroblast-morphology and appear as enlarged cells exhibiting hypertrophy-like features (Figure 1, P7, bottom panel). Additionally, the appearance of intracellular stress fibres was also abundant, with passage numbers of 15 and above (Figure 1, P15 bottom panel). Our bright-field microscopic images of late-passage MSCs suggested replicative aging as the passage number reached 15 and above, as evidenced by our senescence assay.

Tri-lineage differentiation of MSCs

**Figure 2:**
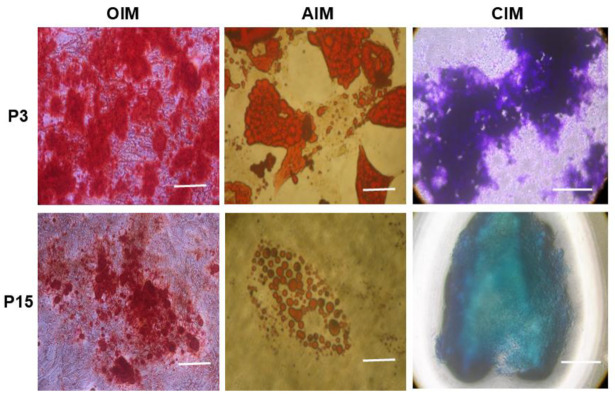
Comparative tri-lineage differentiation potential of early- versus late-passage MSCs. Top panel—bright-field microscopic image of early-passage MSCs (P3) showing profound differentiation potential into osteoblast-like cells (Alizarin red stained), adipocyte-like cells (oil red stained), and chondrocyte-like cells (Toluidine blue stained) (scale bar—10 µm). Bottom panel—bright-field microscopic image of late-passage MSCs (P15 and above) showing diminished differentiation potential into osteoblast-like cells (Alizarin red stained), adipocyte-like cells (oil red stained), and chondrocyte-like cells (Toluidine blue stained) (scale bar—10 µm).

We further investigated the functionality of MSCs with respect to their tri-lineage differentiation potential. We subjected both early-passage MSCs (P3) and late-passage MSCs (P15) to undergo adipogenic, osteogenic, and chondrogenic differentiation. We observed that early-passage MSCs (P3) efficiently differentiated into the aforesaid tri-lineage, as evidenced from the specific biochemical staining for each lineage (Figure 2 P3, top panel). On the other hand, late-passage MSCs (P15) showed a diminished tendency to commit and undergo tri-lineage differentiation potential, as demonstrated by our biochemical staining from each specific lineage (Figure 2, P15 bottom panel).

Gene expression of tri-lineage differentiation

**Figure 3:**
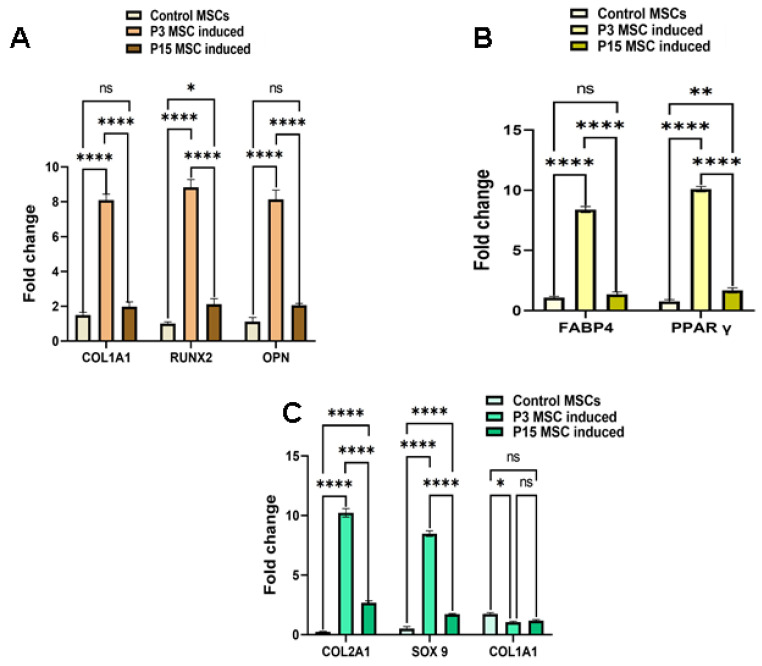
Gene expression profiling of tri-lineage specific genes in early- and late-passage MSCs. (**A**) Bar graph representation of comparative semi-quantitative gene expression profiling of osteoblast-lineage-specific genes. The panel of genes (Collagen type 1, RUNX2, and OPN) were significantly upregulated in early-passage MSCs induced with osteogenic differentiation. The late-passage MSCs showed significantly diminished expression profile, suggesting less efficiency in commitment and undergoing osteoblast lineage. (**B**) Bar graph representation of comparative semi-quantitative gene expression profiling of adipocyte-lineage-specific genes. The panel of genes (FABP4 and PPARγ) were significantly upregulated in early-passage MSCs induced with adipogenic differentiation. The late-passage MSCs showed declined expression profile, suggesting less efficiency in commitment and undergoing adipocyte lineage. (**C**) Bar graph representation of comparative semi-quantitative gene expression profiling of chondrocyte-lineage-specific genes. The Collagen type 2 and Sox9 genes were significantly upregulated with the downregulated expression of Collagen type 1, as evidenced in early-passage MSCs induced with osteogenic differentiation. The late-passage MSCs showed deficit expression profile as compared with early-passage ones, suggesting less efficient in commitment and undergoing chondrocyte lineage. For all statistical analyses, *p* < 0.05 was considered as significant. * *p* < 0.05, ** *p* < 0.01 and **** *p* < 0.0001; ns—non significant.

Our semi-quantitative assay of the tri-lineage specific marker gene expression in the differentiated early- (P3) and late-passage MSCs (P15) also corroborated with our biochemical staining data (Figure 3). We found that osteoblast-specific genes such as Collagen type 1, RUNX2, and OPN were significantly up-regulated in the early-passage (P3) differentiation-induced MSCs, whereas the late-passage differentiation-induced MSCs were found to have a scarce expression of osteoblast-specific genes (Figure 3A). Our data clearly pointed out that the delay in the passage showed a profound effect on the commitment and differentiation of MSCs into osteoblast-like cells.

Similarly, the authors extensively evaluated the biochemical staining and the differentiation property for adipogenic and chondrogenic lineages (Figure 3B,C). We observed a similar pattern, as early-passage MSCs possess an enhanced differentiation potential, as evidenced via biochemical staining and gene expression profiles (FABP4 and PPARγ for adipogenic specific lineage and Collagen type 2 and SOX9 for chondrogenic specific lineage). We concluded that the diminished differentiation potential of MSCs was predominantly incurred due to the aging factor. Several studies have also reported that the differentiating propensity of MSCs diminishes with aging. This is one of the major restricting factors in upscaling MSCs for developing personalised therapeutic applications.

We observed the reversal of morphology, expression of mesenchymal marker vimentin, and differentiation potential in CHIR 99021-treated late-passage MSCs.

**Figure 4:**
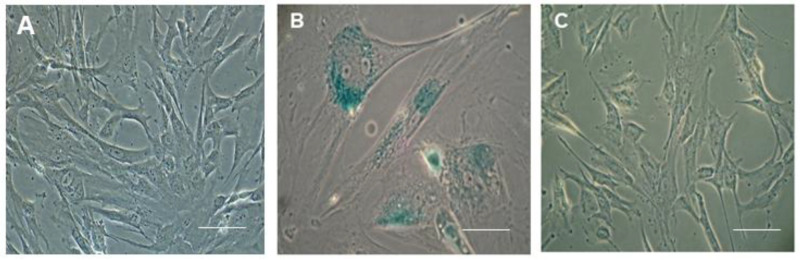
Senescence dye uptake assay. (**A**) P3 MSCs showed no uptake of senescence-specific dye. (**B**) P15 MSCs showed uptake of senescence dye. (**C**) CHIR 99021-treated MSCs showed almost negligible uptake of senescence dye and behaved almost similar to early MSCs. (Scale bar—10 µm.)

**Figure 5:**
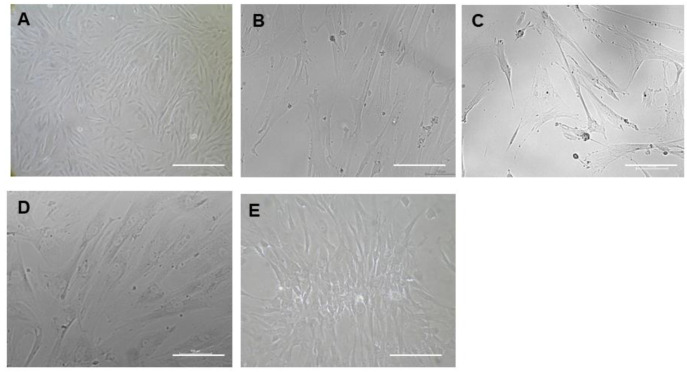
Morphological analysis of CHIR 99021-treated MSCs. (**A**) Phase-contrast bright-field image of typical early-passage P3 MSCs. (**B**) Phase-contrast bright-field image of 72 h post CHIR 99021-treated P15 MSCs showing slender morphology rather than hypertrophic features. (**C**) Phase-contrast bright-field image of post CHIR 99021-treated MSCs after 48 hrs under maintenance medium. (**D**,**E**) Phase-contrast bright-field image of passaged MSCs post treated with CHIR 99021 on day 5 and 7. (Scale bar: 10 µm.)

The effect of CHIR 99021 on MSC senescence is still an unexplored area of research, as CHIR 99021 has been reported to efficiently preserve the pluripotency in ESCs. We were also interested in studying the impact of CHIR 99021 on the late-passage MSCs. Our results were interestingly in favour of rescuing the anti-aging property of MSCs using the Wnt agonist CHIR 99021. We treated MSCs with CHIR 99021 at a concentration of 10 µmolar for 3 consecutive days. In our senescence study, we previously demonstrated that early-passage P3 MSCs failed to ingest the dye, while late-passage MSCs successfully did so following overnight incubation (Figure 4A,B). Interestingly, our study revealed that late-passage MSCs treated with CHIR 99021 had their senescence-specific dye uptake effect reversed, which might be interpreted as a substantial reversal of their senescence-related aging impact (Figure 4C). Upon subjecting the pre-treated MSCs to normal MSC maintenance medium, we could observe the gradual reversion of the MSCs into a nearly fibroblastic shape (Figure 5). The appearance of stress fibres was also reduced (Figure 5). 

**Figure 6:**
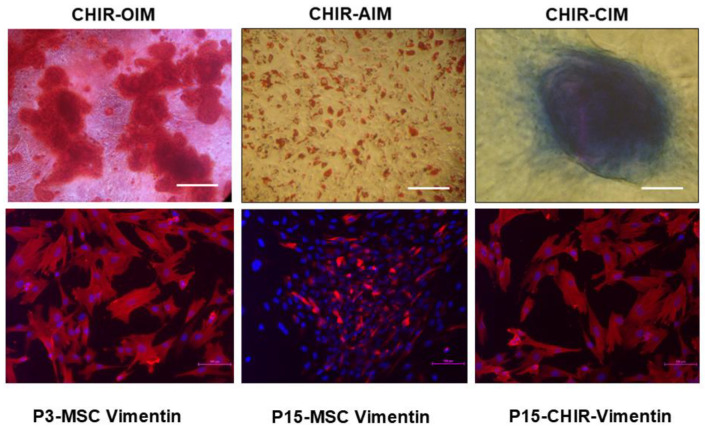
Characterisation of CHIR 99021-treated MSCs for tri-lineage differentiation and typical marker. Top panel—representative bright-field image of biochemical-stained CHIR 99021-treated MSCs for osteoblast, adipocyte, and chondrocyte lineages. Magnification—10×. All the representative bright-field images showed profound staining of corresponding specific features, suggesting significant reversal in functional aspects. Bottom panel—representative ICC image showing vibrant expression of vimentin, a typical MSC marker in early-passage MSCs. Vimentin expression was diminished as the passage number increased significantly (Scale bar—100 μm).

**Figure 7:**
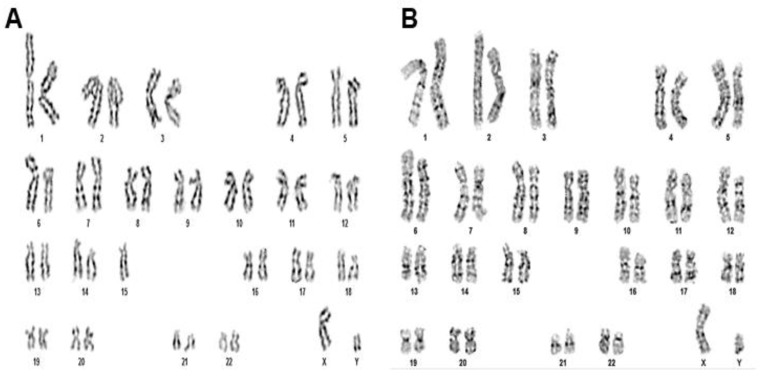
Karyotyping analysis of CHIR 99021-treated late-passage MSCs. (**A**,**B**) Normal karyogram and G banding pattern showing intact 23 pairs of chromosomes in both late-passage MSCs and CHIR 99021-treated late-passage MSCs, suggesting absence of obvious genotoxic effect incurred during CHIR 99021 treatment.

Further, the mesenchymal-specific marker vimentin was found to be diminished in late-passage MSCs (Figure 6, bottom panel). After following the CHIR 99021 pre-treated late-passage MSCs, the expression of vimentin was reversed by showing a higher expression of vimentin compared to untreated late-passage P15 MSCs (Figure 6, bottom). The gene expressions of osteo-specific markers (Collagen type 1, RUNX2, and OPN), adipo-specific (FABP4 and PPARγ) and chondro-specific markers (Collagen type 2 and SOX9) were significantly up-regulated in CHIR-treated late-passage MSCs compared to untreated late-passage MSCs (P15) (Figure 7).

Demonstration of genetic stability by karyotyping

**Figure 8:**
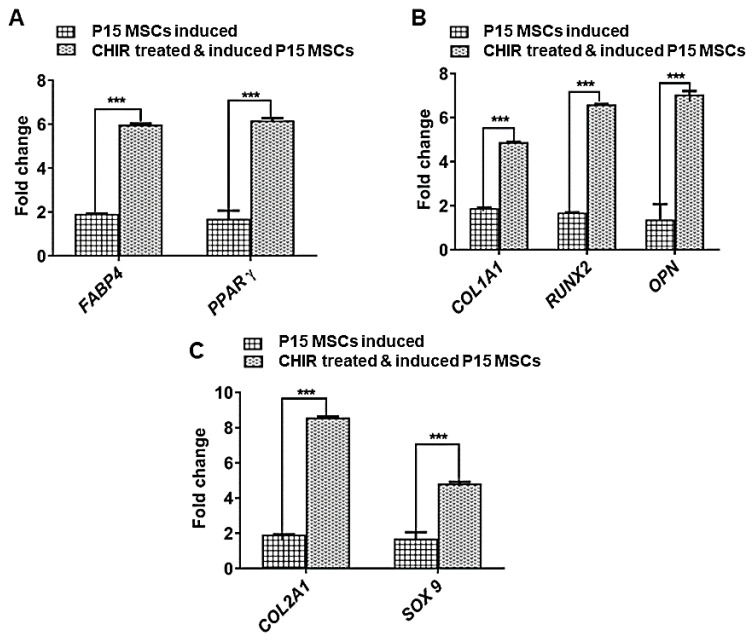
Gene expression profiling of tri-lineage specific genes in late-passage and CHIR 99021-treated differentiation-induced MSCs. (**A**) Bar graph representation of comparative semi-quantitative gene expression profiling of osteoblast-lineage-specific genes. The panel of genes (Collagen type 1, RUNX2, and OPN) were significantly upregulated in CHIR 99021-treated MSCs induced with osteogenic differentiation. However, the untreated MSCs showed diminished expression profile, suggesting after CHIR 99021 treatment a significant reversal in the functional aspects of MSCs. (**B**) Bar graph representation of comparative semi-quantitative gene expression profiling of adipocyte-lineage-specific genes. The panel of genes (FABP4 and PPARγ) were significantly upregulated in CHIR 99021-treated MSCs induced with adipogenic differentiation. However, the untreated MSCs showed diminished expression profile, suggesting after CHIR 99021 treatment a significant setback in the functional aspects of MSCs. (**C**) Bar graph representation of comparative semi-quantitative gene expression profiling of chondrocyte-lineage-specific genes. The panel of genes (Collagen type 2 and SOX9) were significantly upregulated in CHIR 99021-treated MSCs induced with osteogenic differentiation. However, the untreated MSCs showed diminished expression profile for the Collagen type 2 and SOX9 genes. For all statistical analyses, *p* < 0.05 was considered as significant. *** *p* < 0.001.

The genomic stability of chromosomes demonstrated through karyotyping analysis showed that CHIR 99021-treated late-passage P15 MSCs (Figure 8) displayed an intact 44+XY chromosomal banding pattern. Hence, no significant chromosomal abnormalities were observed after treatment.

## 3. Discussion

MSC senescence is a complicated and all-encompassing issue, which severely hampers the therapeutic applications of MSCs. Therefore, improving the delay in senescence and enabling MSCs’ clinical use requires a variety of treatment methodologies and proven protocols. In-depth research on MSC senescence characteristics and its underlying mechanisms via induction, detection, and research on the identification of molecules associated with senescence will aid in the search for methods to rejuvenate senescent MSCs. Independent of tissue source, MSCs undergoing serial passage in culture eventually reach the Hayflick limit, undergoing replicative senescence or in vitro aging [27]. This also depends on the donor’s age, which is essential for MSCs to exhibit unfavourable effects of senescence in vitro. However, low-passage cultures are indicated for the clinical-scale expansion of cultures, since extended passages cause MSCs’ ability to differentiate to deteriorate [28]. Mounting evidence demonstrates that MSCs derived from elderly patients can intensely impair tissue healing applications [29,30]. The aforesaid observation is predominantly due to the wide-ranging difference in the secretory profiles of early- and late-passage MSCs [30]. In order to obtain an abundant, limitless supply of homogenous, stable MSCs for therapy, MSC aging could either be delayed or reversed. This could be achieved by extensively working on avenues for delaying or reversing MSC senescence. This further aids in rejuvenating the patient’s own MSCs and maximising their utilisation to the fullest extent possible. However, a number of limitations prevent the deployment of resurrected MSCs in clinical settings. First, proliferative arrest is consistently attained as the number of passages in the in vitro development of MSCs increases. Collateral impairment could, thus, be caused by the genetic modification of MSCs. The primary physiological routes that regulate MSC senescence play a significant role in regulating common biological processes. Therefore, interventions that entirely repress or activate these pathways may not be appropriate. Therefore, we must balance the treatment’s efficacy against any potential drawbacks.

A hypertrophic morphology develops as a result of changes in biochemical and metabolic properties brought on by aging. MSCs produced from older patients have lower proliferative capacities than those from young, healthy individuals in long-term MSC cultures, regardless of the cell source [31]. MSCs from young donors have higher levels of mitotic activity, a delayed senescence start, and a higher rate of proliferation. In our investigation, we discovered that as the passage number increased to above 15, the WJ-MSCs began to display aging-related characteristics [14,32]. The discrepancy in the onset of the senescence event may vary with the age of the donors and changes in the cultural circumstances, particularly those relating to the serum batch and source [33].

Similar to our study, another group employed CHIR 99021, an aminopyrimidine derivative, and showed the inhibition of GSK-3 in cultured late endothelial progenitor cells (EPCs). The mechanism was found to be via inhibiting the phosphorylation of tuberous sclerosis complex-2 (TSC2), subsequently inhibiting mTOR. Additionally, lysosomal activation and autophagy were significantly boosted when GSK-3 activity was suppressed. In addition to preventing the replicative senescence of late EPCs, GSK-3 inhibition also directed and improved EPCs’ migration, proliferation, and angiogenesis [34]. To sum up, our findings also showed a similar effect in late-passage MSCs, preventing the replicative senescence of MSCs and enhancing their differentiation function. Therefore, based on our findings, CHIR 99021 treatment may offer insight into the management of reversing age-related damage in MSCs. 

Interestingly, another study by Khanh et al., 2020 [32], unambiguously demonstrated rescuing old MSCs’ ROS build up and cellular senescence through treatment with the antioxidant Edaravone or the co-overexpression of superoxide dismutase 1(SOD1) and SOD3, improving their activities. Further, human Wharton’s jelly-MSC-derived EVs rejuvenated older MSCs by preventing ROS generation and promoting proliferation and in vivo functions in both type 1 and type 2 diabetic mice. Another study demonstrated that human umbilical-cord-derived MSC–Extracellular Vesicles (UC-EVs) enriched anti-aging signals and regenerated senescent adult bone-marrow-derived MSCs by transferring proliferating cell nuclear antigen (PCNA) into aged MSCs [35]. Further, UC-EV-rejuvenated MSCs showed reduced aging characteristics with an increasing self-renewal capability and telomere length. Our study was in line with MSC rejuvenation; however, we employed a WNT agonist for rescuing the functional aspects of MSCs. Thus, the use of revitalised MSCs from the bench to the bedside is still constrained by a number of the abovementioned factors, which need to be thoroughly addressed before application in clinical settings.

## 4. Materials and Methods

### 4.1. Isolation of WJ-MSCs

After informed consent, human umbilical cord tissue (hUCT) samples were collected from mothers undergoing the C-section mode of delivery. The samples were collected aseptically in Dulbecco’s Phosphate-Buffered Saline (DPBS) containing 1× antibiotic and anti-mycotic solutions (Gibco, Thermo Fisher Scientific, Waltham, MA, USA) and processed within 24 h of collection by following the protocol established by Takahashi-Yanaga et al., with few modifications [26]. Cells were seeded at a density of 1 × 10^7^ nucleated cells/mL in 25 cm^2^ flask with complete medium (α-MEM supplemented with 10% FBS). Then, cultures were incubated at 37 °C in a humidified CO_2_ incubator (Forma^TM^ Steri-Cycle^TM^ CO_2_ incubator, Thermo Scientific, Waltham, MA, USA). The culture medium was changed at every 2 days interval until 80% confluency was reached. Briefly, upon reaching confluency, cells were harvested using trypsin enzyme (Gibco, Thermo Fisher Scientific, Waltham, MA, USA) and propagated at a ratio of 1:3. MSCs were progressively passaged up to number 17 and above under the abovementioned culture conditions and were used for the experimental analysis.

### 4.2. Pre-Treatment of WJ-MSCs with CHIR 99021

The modified protocol demonstrated by Govarthanan et al. was used to treat the late-passage MSCs [4]. Briefly, passage number 15 and above WJ-MSCs were treated with a medium composed of MEM medium supplemented with 1× Insulin-transferrin-sodium selenite, 50 μg/mL L-ascorbic acid 2-phosphate, 0.1 mM β-mercaptoethanol, 2 mM Glutamax, 1% non-essential amino acids (Gibco, Thermo Fisher Scientific, Waltham, MA, USA), and 10 µM CHIR 99021 (Sigma Aldrich, St. Louis, MO, USA) and incubated at 37 °C in a 5% humidified CO_2_ incubator (Forma^TM^Steri-Cycle^TM^ CO_2_ incubator, Thermo Scientific, Waltham, MA, USA) for 72 h. After the treatment, the MSCs were cultured in a normal maintenance medium made of MEM and supplemented with 10% FBS. All the assays were conducted in the CHIR 99021-treated cells as obtained from the aforesaid methods and characterised further.

### 4.3. Tri-Lineage Differentiation Potential of MSCs

Early-passage MSCs (P3), late-passage MSCs (P15), and late-passage MSCs (P17) treated with CHIR 99021 were induced with an osteogenic induction medium composed of α-MEM medium supplemented with 10% FBS, 100 nM dexamethasone (Sigma Aldrich, St. Louis, MO, USA), 10 mM β-glycerol phosphate (Sigma Aldrich, St. Louis, MO, USA), and 50 μM ascorbate-2-phosphate (Sigma Aldrich, St. Louis, MO, USA). Mineralisation was studied by Alizarin Red S staining (Himedia, Maharashtra, India) after 21 days and osteoblast-specific genes were quantified by qPCR using an Applied Biosystems 7500 Real-Time PCR instrument (Applied Biosystems, Thermo Fisher Scientific, Waltham, MA, USA) for analysing osteoblast differentiation [4].

Early-passaged MSC (P3), late-passage MSCs (P15), and MSCs treated with CHIR 99021 (P17) were induced with an adipocyte induction medium composed of MEM medium supplemented with 10% FBS, 1 μM dexamethasone, 0.5 mM isobutyl-methyl xanthine (IBMX), 100 ng/mL insulin, and 60 μM indomethacin (Sigma Aldrich, St. Louis, MO, USA). The cells were incubated for next 28 days at 37 °C in a 5% humidified CO_2_ incubator (Forma^TM^Steri-Cycle^TM^ CO_2_ incubator, Thermo Scientific, Waltham, MA, USA) by changing the media twice in a week. Adipocyte differentiation was confirmed with Oil Red O staining (Sigma Aldrich, St. Louis, MO, USA) and quantitatively analysed by qPCR using an Applied Biosystems 7500 Real-Time PCR instrument (Applied Biosystems, Thermo Fisher Scientific, Waltham, MA, USA) [4].

For chondrocyte induction, early-passage MSCs (P3), late-passage MSCs (P15), and MSCs treated with CHIR 99021(P17) were cultured in chondrocyte differentiation medium (Gibco, Thermo Fisher Scientific, Waltham, MA, USA) as a pellet culture system and incubated for next 28 days at 37 °C in a 5% humidified CO_2_ incubator (Forma^TM^Steri-Cycle^TM^ CO_2_ incubator, Thermo Scientific, Waltham, MA, USA) by changing the media on every 3rd day. Chondrocyte differentiation was studied by toluidine blue staining (Sigma Aldrich, St. Louis, MO, USA) and quantitatively analysed by qPCR using an Applied Biosystems 7500 Real-Time PCR instrument (Applied Biosystems, Thermo Fisher Scientific, Waltham, MA, USA) [4].

### 4.4. Senescence Assay

Senescence assay was performed as per the manufacturer’s protocol given by the Senescence β-Galactosidase Staining kit (Cell signaling technology, Danvers, MA, USA). Briefly, cells seeded in 6-well plates were washed with PBS and fixed with the fixative solution provided in the kit. The fixed cells were washed with PBS twice, followed by the addition of 1 mL of the β-Galactosidase Staining Solution to each well. The plate was sealed with parafilm and allowed to incubate overnight at 37 °C in a dry incubator. The next day, cells were observed under the microscope for the uptake of blue dye, which indicates senescent cells.

### 4.5. G-Banded Karyotyping

G-banding karyotyping was performed as per the protocol followed by Vennila et al. [36]. At 80% confluency, colcemid (10 mg/mL) was added at a final dilution of 0.1 µg/mL and incubated at 37 °C for 30 min. Morphological changes were observed under a microscope. After trypsinization, cells were centrifuged at 400× *g* for 10 min, followed by hypotonic treatment. Hypotonic treatment was performed as follows: 10 mL of 0.075 M KCl was added slowly, incubated at 37 °C for 30 min, and fixed with Carnoy’s fixative (methanol: acetic acid in 3:1) solution. Cells were allowed to burst to obtain the chromosome spread by dropping the cell suspension onto a precleaned cooled slide from a height for the uniform blast. The slides were baked at 80 °C for 1 h and then placed at 37 °C for at least 16 hr, immersed in trypsin solution (0.002 g/mL) for 5 s, washed in Sorenson’s buffer, and finally quickly rinsed in distilled water. Staining was performed using Giemsa dye (1: 20). Trypsin and Giemsa (GTG) bands were studied by microscopy (100×). At least 20 metaphases were analysed using Cytopower from Applied Spectral Imaging (ASI) software version 3 to obtain the karyogram.

### 4.6. RNA Extraction and cDNA Synthesis

Total RNA was extracted from early-passage P3 MSCs, late-passage P15 MSCs, and P15 MSCs treated with CHIR 99021 using the Trizol method (Sigma Aldrich, St. Louis, MO, USA) according to the manufacturer’s recommended protocol. Approximately 2 µg of total RNA was reverse-transcribed into cDNA in a 20 µL reaction volume using MMLV-RT enzyme (Thermo Fisher Scientific, Waltham, MA, USA) and oligo-dT primers (New England Biolabs, Ipswich, MA, USA).

#### Gene Expression Analysis

Semi-quantitative Real-Time PCR (RT-PCR) was performed with corresponding primers of the specific cell types listed in Table S1. Briefly, a two-step cycling protocol was performed, consisting of a single 10 min cycle at 95 °C, followed by 40 cycles of 10 s at 95 °C, and 30 s at 60 °C. The melting curve analysis was performed after amplification to ensure the specificity of the products. The relative mRNA expression was quantified by normalising with housekeeping gene β-actin, then fold change was calculated by ΔΔct method.

### 4.7. Immunocytochemistry (ICC)

ICC analysis was performed in early-passage P3 MSCs, late-passage P15 MSCs, and P15 MSCs treated with CHIR 99021 for assessing the expression of vimentin, a mesenchymal marker. Cells were fixed with 2% paraformaldehyde solution for 40 min at room temperature and permeabilised with 0.25% Triton X-100 in 1 × PBS solution for 15 min. The fixed cells were washed with DPBS three times and incubated with a blocking buffer containing 5% normal goat serum for 1 h at room temperature. Further, the cells were incubated with vimentin primary antibody overnight at 4 °C with gentle shaking. Then, the cells were washed with DPBS and incubated again for the next 2 h with the appropriate secondary antibody (IgG Alexa flor-594 with 1:1000 dilutions) at room temperature. Nuclei were stained with Hoechst 33,258 dye (Sigma Aldrich, St. Louis, MO, USA). The stained cells were observed under a fluorescence microscope (Nikon Tie, Tokyo, Japan) at 10× magnification.

### 4.8. Statistical Analysis

All experiments were performed in biological triplicates (*n* = 3). The statistical analysis was performed by one way and two-way ANOVA tests using graph pad prism 8.0 software. For multiple comparisons, Turkey’s multiple comparison test was used. For all statistical analyses, *p* < 0.05 was considered as significant. * *p* < 0.05, ** *p* < 0.01, *** *p* < 0.001 and **** *p* < 0.0001.

## 5. Conclusions

In summary, the present study demonstrated that the age of late-passage MSCs can be significantly reversed and the functional potential of MSCs with respect to tri-lineage differentiation can be extensively restored by pre-treating with the WNT agonist CHIR 99021. Forthcoming studies on the in-depth mechanisms of the molecular pathways may provide us with the mechanistic aspects of rejuvenation while pursuing clinical applications. Furthermore, standard operating procedures may be established to ensure that MSCs derived from shell products are devoid of such pre-treatment chemicals before being delivered to patients.

## Data Availability

The datasets generated and analysed during the current study are available from the corresponding author (R.S.V.) upon reasonable request.

## Supplementary Materials

The following supporting information can be downloaded at: https://www.mdpi.com/article/10.3390/ph18040483/s1, Table S1: List of Primers used in the study.
