# Supplementary material for: Inhibition of GSK-3β Restores Differentiation Potential of Late-Passage Mesenchymal Stem Cells
Source: Pharmaceuticals (Basel). 2025 Mar 28;18(4):483. doi: 10.3390/ph18040483 (PMC12030495; doi:10.3390/ph18040483)
Supplement: Supplementary file 1 [file pharmaceuticals-18-00483-s001.zip › pharmaceuticals-3514523-Supplementary.pdf]

**Table S1.** List of qRT-PCR Primers used in the study.

| S No | Gene | Primer Sequence |
|---|---|---|
| 1 | *SOX9* | FP-AGCACTCATAATATGGCATCCTTCA<br>RP- AGGTAAGTTTCACGGAGAGAACAA |
| 2 | *RUNX2* | FP-GCGGTGCAAACTTTCTCCAG<br>RP-TCACTGTGCTGAAGAGGCTG |
| 3 | *Collagen Type 1* | FP- GGCCATCCAGCTGACCTTCC<br>RP- CGTGCAGCCATCGACAGTGAC |
| 4 | *Collagen Type 2* | FP- TGAACGAGGTTTCCCAGGTG<br>RP- CCAGGCATTCCCTGAAGACC |
| 5 | *OPN* | FP-TTGCAGCCTTCTCAGCCAA<br>RP- GGAGGCAAAAGCAAATCACTG |
| 6 | *PPAR gamma* | FP- AGGCGAGGGCGATCTTGACAG<br>RP- GATGCGGATGGCCACCTCTTT |
| 7 | *FABP4* | FP-GCTTTGCCACCAGGAAAGTG<br>RP- ATGGACGCATTCCACCACCA |
